# Antibodies reacting with JCPyV_VP2 _167-15mer as a novel serological marker for JC polyomavirus infection

**DOI:** 10.1186/1743-422X-11-174

**Published:** 2014-10-01

**Authors:** Ole Lagatie, Tom Van Loy, Luc Tritsmans, Lieven J Stuyver

**Affiliations:** Janssen Diagnostics, Turnhoutseweg 30, 2340 Beerse, Belgium; Janssen Research and Development, Turnhoutseweg 30, 2340 Beerse, Belgium

**Keywords:** JC Polyomavirus, Biomarker, Peptide serology, VP2, Progressive Multifocal Leukoencephalopathy

## Abstract

**Background:**

JC polyomavirus (JCPyV) is a widespread human polyomavirus that usually resides latently in its host, but can be reactivated under immune-compromised conditions potentially causing Progressive Multifocal Leukoencephalopathy (PML). Detection of antibodies against the major capsid protein VP1 currently is the main marker for assessment of infection with JCPyV.

**Methods:**

Based on a peptide microarray, peptide JCPyV_VP2_167-15mer was selected and a peptide ELISA was developed for detection of antibodies directed against this peptide. Epitope mapping and computational modelling was performed to further characterize this peptide. In a cohort of 204 healthy subjects it was investigated whether antibodies against JCPyV_VP2_167-15mer were correlated with VP1 serology or urinary viral load.

**Results:**

Epitope mapping of peptide JCPyV_VP2_167-15mer showed that the minimal epitope consisted of L_173_PALTSQEI_181_ with amino acids P_174_, L_176_ and E_180_ being essential for antibody recognition. Computational analysis was used to predict that this epitope is located at an exposed domain of the VP2 capsid protein, readily accessible for immune recognition upon infection. No correlation could be observed with JCPyV VP1 antibody levels, or urinary viral load.

**Conclusion:**

This work indicates that specific antibodies against JCPyV_VP2_167-15mer might be considered as a novel serological marker for infection with JCPyV.

**Electronic supplementary material:**

The online version of this article (doi:10.1186/1743-422X-11-174) contains supplementary material, which is available to authorized users.

## Introduction

JC Polyomavirus (JCPyV) is a human neurotropic polyomavirus that was found to be the causative agent of progressive multifocal leukoencephalopathy (PML), a fatal demyelinating disease [1–4]. JCPyV can switch from its latent state to an activated state in immunocompromised subjects such as HIV-1 infected patients and in multiple sclerosis (MS) patients treated with natalizumab [5–7]. The JC Polyomavirus capsid is composed of 72 pentamers of the major capsid protein VP1, with one of the minor coat proteins (VP2 or VP3) in the center of each pentamer Both minor proteins are essential for the viral life cycle [8, 9] and were shown to act as membrane proteins during infection and to form pores in host cell membranes [10].

Antibodies to JCPyV VP1 are widely prevalent in healthy subjects indicating that most individuals have been exposed to or are latently infected with the virus [11–17]. Antigenic epitopes have been described for VP1, with most of these epitopes being shared between JCPyV and the other polyomaviruses BKPyV and SV40 [18]. Despite this epitope sharing, JCPyV specific serology assays using full length recombinant JCPyV VP1 as antigen were developed. Specificity was shown by inhibition experiments using VP1 from other known polyomaviruses [19–21]. Serological results should however be interpreted with caution as serological cross-reaction with closely related, yet unidentified human polyomaviruses can never be excluded [22–24]. Currently, the STRATIFY JCPyV ELISA using baculovirus-expressed VP1 virus-like-particles (VLP) as antigen, is the only Food and Drug Administration (FDA) approved assay for JCPyV [12, 25].

Little attention has been paid so far to JCPyV VP2 or VP3 as immunogenic proteins, although some examples have been described of the immunogenic nature of these minor capsid proteins in other polyomaviruses. A high prevalence of antibodies against VP2 has been described for WU Polyomavirus [26]. Furthermore, a linear epitope was identified in SV40 VP2/VP3 that showed immunoreactivity in serum from 21.9% of blood donors [27]. Also BKPyV VP2 and VP3 were identified as targets of cellular immunity [28]. Peptide microarray analysis using a comprehensive set of polyomavirus derived peptides demonstrated that several non-VP1 peptides were recognized by antibodies in human plasma and could potentially represent linear epitopes of these proteins [29].

In this work we have investigated, using a peptide microarray setup, whether linear epitopes could be identified in JCPyV VP2. A 15-mer peptide was identified that was thereafter used for the development of a peptide ELISA. The immunoreactivity of this peptide was further characterized and its relationship with other JCPyV markers was investigated.

## Results and discussion

**Figure 1:**
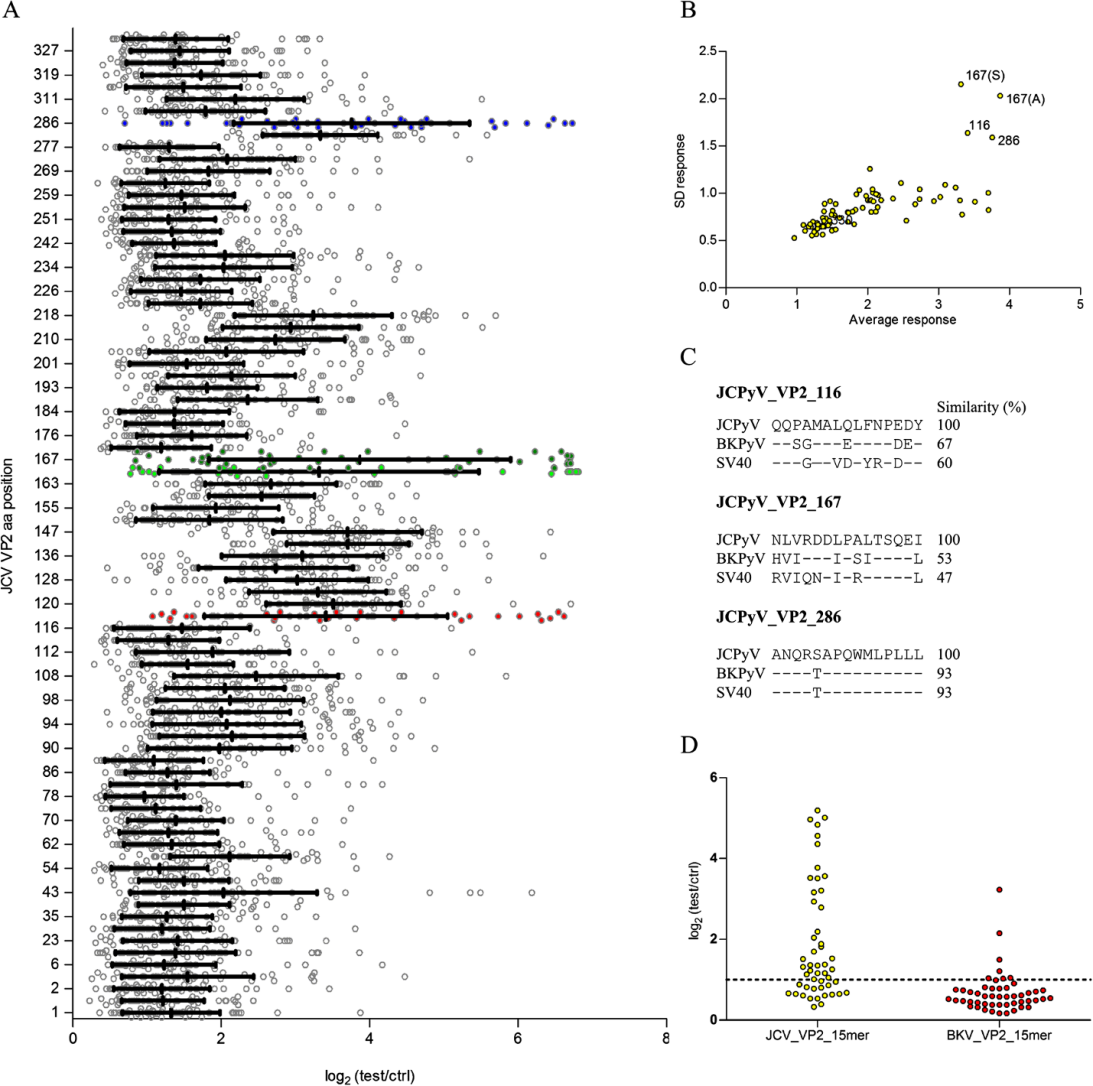
**Identification of JCPyV_VP2_167-15mer as immunoreactive peptide. (A)** Distribution of the JCPyV VP2 polyomavirus 15-mer peptide microarray signals obtained with plasma samples from 49 HSs, diluted at 1:200. Peptides with the largest signal distribution are indicated in red (JCPyV_VP2_116-15mer), green (JCPyV_VP2_167-15mer, light green is S_175_ variant, dark green is A_175_ variant) and blue (JCPyV_VP2_286-15mer). **(B)** Average signal in microarray vs. standard deviation of signal over the different subjects, plotted per peptide. **(C)** Similarity between peptides specific to JCPyV, BKPyV and SV40 for the 3 selected JCPyV peptides. **(D)** Plasma antibody reactivity of 50 HSs against JCPyV_VP2_167-15mer and BKPyV_VP2_167-15mer, with data presented as log_2_ (signal of test sample / signal of the no-sample control). Samples were diluted at 1:200 and detected in a peptide ELISA.

A total of 82 15-mer peptides derived from JCPyV VP2 were incubated in a peptide microarray format with plasma samples from 49 healthy subjects (HS), resulting in 4018 data points. All individual data points were plotted per peptide and the mean value and standard deviation was calculated per peptide (Figure 1A). Upon plotting these descriptive statistics on an x-y plot, 4 peptides clearly had different responses compared to the other peptides, with high average response and high variation over the different subjects (Figure 1B). These peptides were JCPyV_VP2_116-15mer, JCPyV_VP2_167-15mer (variant with S_175_ and variant with A_175_) and JCPyV_VP2_286-15mer. Remarkably, two variants of the same peptide (JCPyV_VP2_167-15mer) both had similar results, suggesting that the variant position is not involved in the epitope recognition. Since JCPyV VP2 is highly homologous to BKPyV VP2 and SV40 VP2, sequence similarity was assessed between the identified JCPyV peptides and the corresponding peptides in BKPyV and SV40 (Figure 1C). For peptide JCPyV_VP2_167-15mer this analysis showed that the corresponding SV40 peptide overlaps largely with a peptide that was identified earlier as an epitope that is recognized by antibodies in serum samples from healthy donors carrying SV40 [27]. As there is also low sequence homology of this peptide with the corresponding BKPyV and SV40 peptides, we decided to further investigate this specific JCPyV peptide. In order to verify that human plasma samples contain IgG antibodies that react with this specific peptide, a peptide ELISA was set up using the synthetic peptides JCPyV_VP2_167-15mer and BKPyV_VP2_167-15mer. This ELISA was used to test for anti-peptide antibody responses in plasma from 50 HSs that had been diluted 200-fold (Figure 1D). This confirmed that plasma from healthy individuals indeed contains IgG antibodies reacting with this peptide, with an overall prevalence of 64% (32/50) for JCPyV_VP2_167-15mer and only 14% (7/50) for BKPyV_VP2_167-15mer.

**Figure 2:**
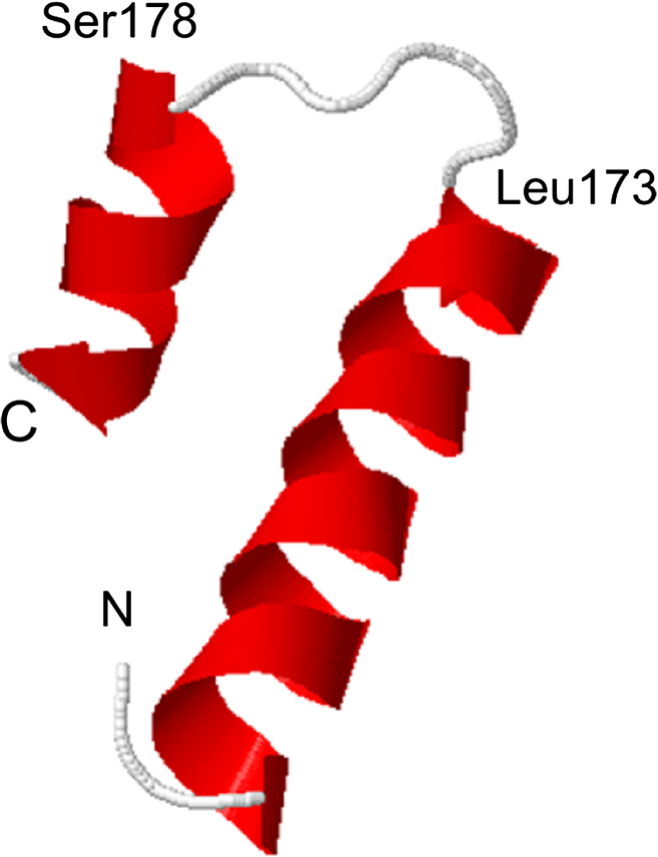
**Secondary structure visualization (PEP-FOLD) of the results of the computational analysis carried out on synthetic polypeptide JCPyV_VP2_154_36-mer.**

Molecular modelling was performed on a 36-mer, covering amino acids 154 to 189 of JCPyV VP2 (Figure 2). This modelling predicts that this peptide is characterized by a stable secondary structure consisting of 2 α helix domains flanking a smaller exposed amino acid stretch. This computational modelling indicates that the majority of JCPyV_VP2_167 is located in an exposed region of the VP2 protein and as such this physical configuration might favour its immunological reactivity, in line with the results observed in the peptide ELISA.

**Figure 3:**
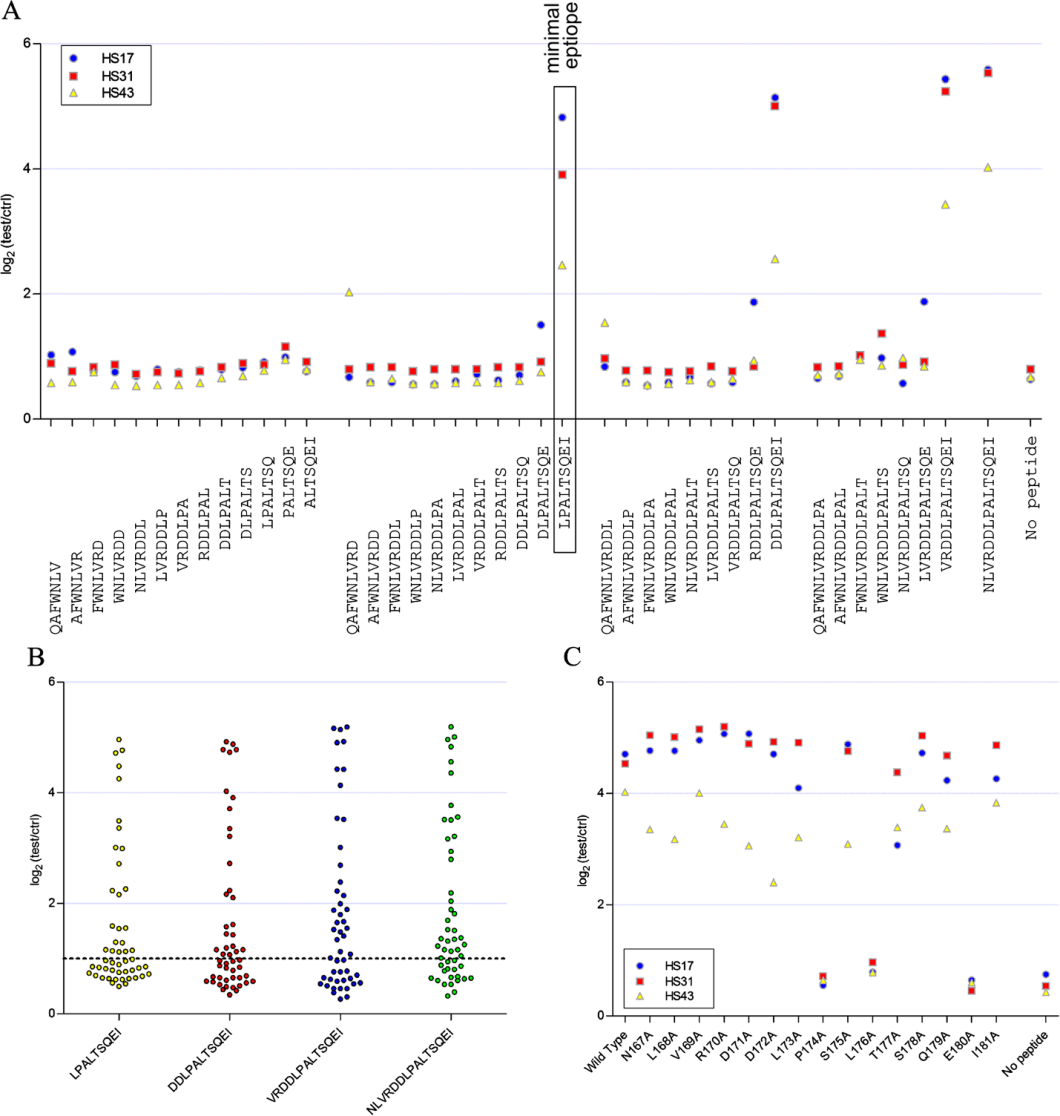
**Epitope mapping of JCPyV_VP2_167-15mer. (A)** Analysis of 7-mers, 9-mers, 11-mers and 13-mers. Plasma antibody reactivity of plasma samples from 3 HSs (diluted 1:200) with high immunoreactivity against the wild type peptide was determined. **(B)** Plasma antibody reactivity of 50 HSs against JCPyV_VP2_173-9mer, JCPyV_VP2_171-11mer, JCPyV_VP2_169-13mer and JCPyV_VP2_167-15mer. Samples were diluted at 1:200 and detected in a peptide ELISA. **(C)** Alanine walking of JCPyV_VP2_167-15mer. Plasma antibody reactivity of plasma samples from 3 HSs (diluted 1:200) with high immunoreactivity against the wild type peptide was determined.

In order to better characterize the immunoreactivity of this JCPyV VP2 derived peptide, epitope mapping experiments were performed. For this investigation, a peptide ELISA was employed to test 3 plasma samples that were highly reactive towards JCPyV_VP2_167-15mer using different specifically designed synthetic peptides. On the one hand a series of 7-mers, 9-mers, 11-mers and 13-mers, overlapping with all but one residue was tested (Figure 3A). This test showed that a minimal peptide required to retain immunoreactivity consisted of the sequence LPALTSQEI. This observation was confirmed by the analysis of anti-peptide antibody responses in plasma from 50 HSs using the 9-mer, 11-mer, 13-mer and 15-mer that was found to retain immunoreactivity (Figure 3B). On the other hand each of the amino acids in JCPyV_VP2_167-15mer was replaced individually by alanine (alanine walking) (Figure 3C). Using this approach the contribution of each specific residue used to the immunoreactivity of the peptide is determined [30, 31]. The data obtained using these peptides in the peptide ELISA indicate that three residues in JCPyV_VP2_167-15mer are important for antibody recognition: P_174_, L_176_ and E_180_. Replacement of any of these residues by alanine resulted in the complete elimination of immunoreactivity of the peptide. These data also confirmed the results of the previous experiment as all essential amino acid residues are located within the minimal peptide affirmed. Remark that the corresponding BKPyV peptide (IPSITSQEL) has an isoleucine residue at position 176 instead of leucine.

Since several mutations have been described in the VP2 region of JCPyV, it might be of interest to investigate whether some of these mutations are located in the JCPyV_VP2_167-15mer. A first mutation that is known, is the E_172_ mutation [32]. However, this residue is not located in the minimal epitope and as such is unlikely to play a role in antibody recognition. A second mutation is the S_175_ mutation which was already part of our initial investigation on the peptide array. Based on both the array data and the epitope mapping data, it is clear that this residue also does not play a role in antibody recognition. Taken together, we might conclude that the peptide ELISA using the JCPyV_VP2_167-15mer NLVRDDLPALTSQEI is capable of detecting antibodies against all JCPyV strains, independent of the strain specific sequence of this VP2 region.

**Figure 4:**
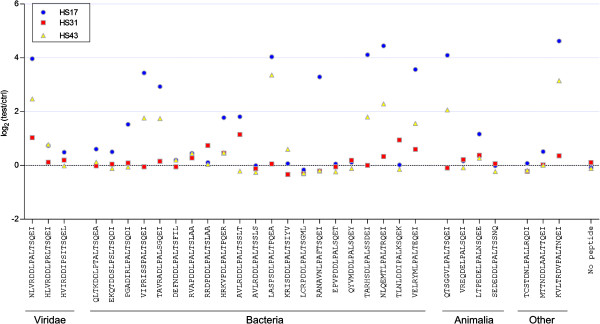
**Plasma antibody reactivity of plasma samples from 3 HSs (diluted 1:200) with high immunoreactivity against JCPyV_VP2_167-15mer was determined using peptides derived from BLAST analysis of JCPyV_VP2_167-15mer (Additional file** 1 **: Table S1).**

The data presented above are demonstrative of the immunoreactivity of JCPyV_VP2_167-15mer and its minimal epitope LPALTSQEI. This does however not imply that the immune responses observed are elicited by exposure to or infection with JCPyV. Any protein containing the LPALTSQEI sequence or closely related sequence might in fact be the antigen responsible for the positive immune responses observed. The responses observed with JCPyV_VP2_167-15mer would then be non-specific responses. In order to investigate the possibility that a protein from another organism might be at the basis of the observed responses, a protein BLAST search was performed against the non-redundant protein database using JCPyV_VP2_171-11mer as query sequence [33] (Additional file 1: Table S1). Within the Viridae only SA12 VP2 and BKPyV VP2 were identified, respectively with 1 or 4 mismatches compared to the query sequence. Also in the human proteome no proteins were identified with less than 4 mismatches. Most proteins with less than 4 mismatches compared to JCPyV_VP2_171-11mer, however, were found in Bacteria and Animalia. Also one protein from protozoan origin and one protein from a grass species were identified. Of all identified sequences the corresponding 15-mer peptide was synthesized and used for assessment of their immunoreactivity in 3 plasma samples that were highly reactive towards JCPyV_VP2_167-15mer. Out of the 30 peptides tested, 9 showed some response in the peptide ELISA that were similar as JCPyV_VP2_167-15mer (Figure 4). The strongest signals were observed for isopropylmalate isomerase from Micrococcus luteus, anti-FecI sigma factor FecR from Spirosoma linguale DSM 74, hypothetical protein from Beggiatoa alba, putative perixosomal biogenesis factor from Ixodes scapularis and probable E3 ubiquitin-protein ligase ARI1-like from Brachypodium distachyon. Although it cannot be excluded, it is very unlikely that one of these proteins is responsible for the immune response observed in the peptide ELISA using JCPyV_VP2_167-15mer. First of all some of these organisms are either rare or occur only at specific locations. Secondly, the proteins identified are not expected to be strong antigens as they are known to be located intracellularly and as such are unlikely to be exposed to the host’s humoral immune system. All together, these data suggest that an immune response against JCPyV VP2 can be observed in HSs and that the amino acid stretch with sequence LPALTSQEI functions as an epitope on this protein.

**Figure 5:**
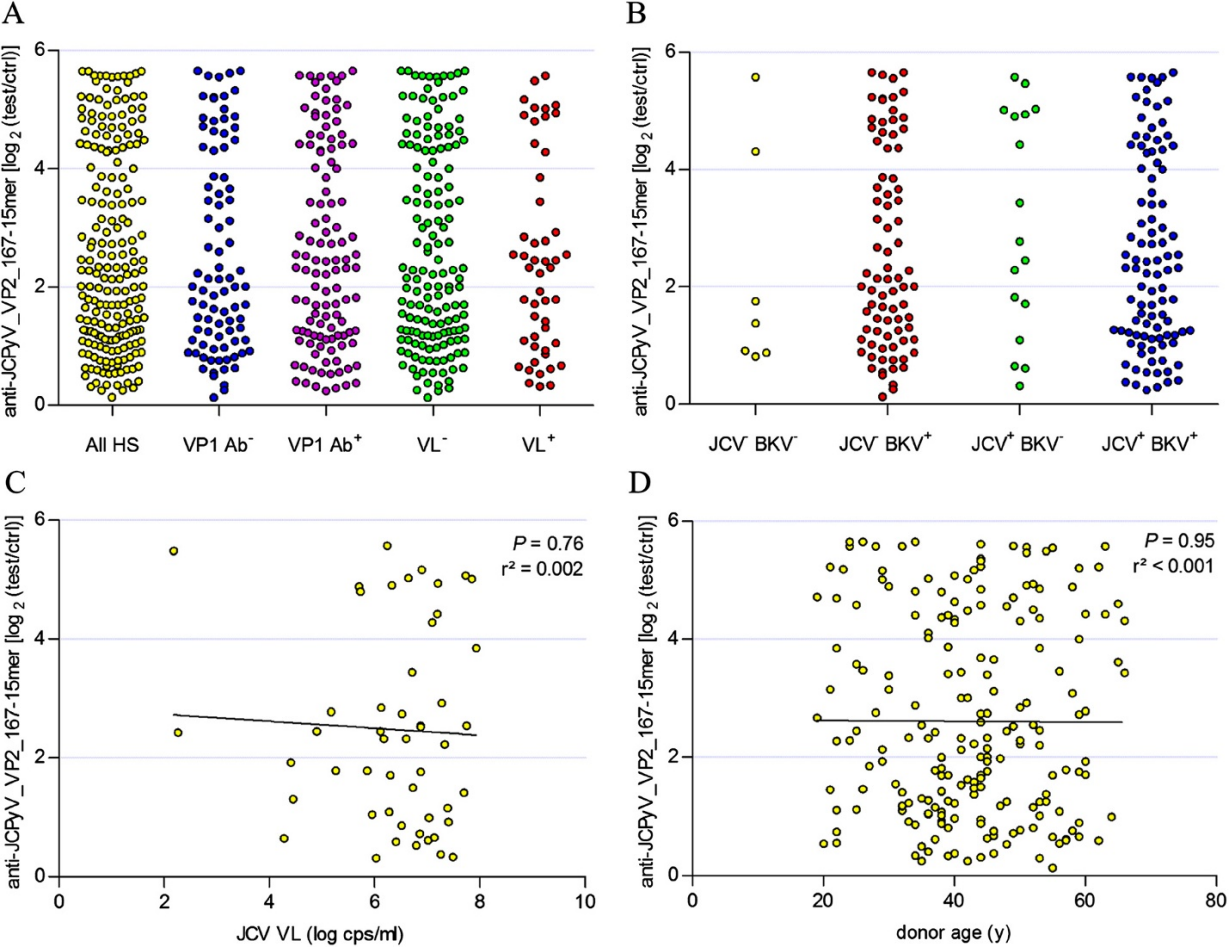
**Plasma antibody reactivity of 204 HSs against JCPyV_VP2_167-15mer. (A)** Subjects were grouped based on JCPyV VP1 serostatus (VP1 Ab^-^ and VP1 Ab^+^) and on urinary viral load (VL^-^ and VL^+^) and antibody reactivity against JCPyV_VP2_167-15mer was determined in the different groups. **(B)** Subjects were grouped based on JCPyV VP1 serostatus and BKPyV VP1 serostatus (JCV^-^BKV^-^, JCV^-^BKV^+^, JCV^+^BKV^-^ and JCV^+^BKV^+^) and antibody reactivity against JCPyV_VP2_167-15mer was determined in the different groups. **(C)** Correlation analysis of plasma antibody reactivity against JCPyV_VP2_167-15mer and JCPyV urinary viral load in HSs. **(D)** Correlation analysis of plasma antibody reactivity against JCPyV_VP2_167-15mer and age.

Based on these data, we believe JCPyV_VP2_167-15mer truly acts as an epitope and therefore the peptide ELISA using JCPyV_VP2_167-15mer as antigen was employed to analyse the immune response against this peptide in a larger cohort of 204 HSs (Figure 5). As was already seen in the smaller cohort of 50 HSs, there was a large variation over the different subjects. Subjects were further categorized based on their JCPyV VP1 serology and JCPyV urinary viral load (Figure 5A). However, no difference (*P* > 0.05) between JCPyV VP1 seropositive and seronegative subjects could be observed. The same was observed when comparing subjects shedding virus in their urine and virus with a negative urinary viral load. Although we have shown before there is no cross-reactivity with BKPyV, we investigated the possibility that infection with BKPyV was interfering in the assay. Therefore, also BKPyV VP1 antibody levels were determined and subjects were categorized based on their JCPyV VP1 and BKPyV VP1 serology (Figure 5B). Again, no difference (*P* > 0.05) was observed between any of the groups. It is known urinary viral load and JCPyV VP1 antibody levels are correlated [34]. We have investigated whether this is also true for JCPyV_VP2_167 antibody levels (Figure 5C). In contrast to VP1 antibody levels, no correlation was found between urinary viral load and JCPyV_VP2_167 antibodies (*P* > 0.05). It was also apparent that JCPyV_VP2_167 antibody levels were not correlated with donor age (*P* > 0.05, Figure 5D). It will also be of interest for future studies to investigate whether JCPyV_VP2_167 antibody levels remain constant over time as is the case for VP1 antibody levels [35, 36].

The lack of correlation between JCPyV_VP2_167 and JCPyV VP1 antibody levels is an interesting, but surprising finding. It is however not the first time that JCPyV VP1 serology is debated for its use as the sole infection marker as several examples have been described where JCPyV DNA was detected in urine, plasma or specific blood cell populations of individuals who were seronegative for JCPyV VP1 [37–40]. In recent work, also a lack of correlation was observed between the level of anti-JCPyV antibody and anti-JCPyV neutralizing activity as 28.6% of JCPyV VP1 Ab^+^ samples were not capable of neutralizing the *in vitro* infection of COS7 cells by JCPyV [41]. Further research would however be needed to investigate whether JCPyV_VP2_167 antibodies are capable of neutralizing JCPyV infection.

It might be debated whether JCPyV VP2 can be recognized by the immune system as it was proposed that VP2 is located at the inner side of the capsid [42]. Crystal structure analysis however showed that the N-terminal part of VP2, including the JCPyV_VP2_167-15mer region is not tightly folded and retains high flexibility, making it easier for VP2 to emerge from inside the virion [43, 44]. The fact that it was also shown that 52.3% of serum samples show immunoreactivity with WU Polyomavirus VP2 and 21.9% of blood donors react with SV40 VP2 derived peptides, strengthens the conclusion that JCPyV VP2 acts as an antigen recognized by the immune system upon JCPyV infection [26, 27]. We postulate that the peptide studied in this work, JCPyV_VP2_167-15mer, acts as one of the epitopes responsible for the antigenic properties of JCPyV VP2.

## Conclusions

The work presented here shows that plasma from a large portion of HSs reacts specifically with a peptide derived from JCPyV VP2 capsid protein. Epitope mapping experiments demonstrated that the minimal epitope consisted of L_173_PALTSQEI_181_ with amino acids P_174_, L_176_ and E_180_ essential for antibody recognition. Computational analysis was used to show that this epitope is located at an exposed domain of the VP2 capsid protein, readily accessible for immune recognition upon infection. Furthermore, this peptide is also located in a region homologous to an immunoreactive domain in SV40 VP2 [27]. BLAST analysis of this peptide and subsequent experimental analysis of the obtained peptides showed that a number of alternative proteins might exist that could have elicited the observed immune responses. However, based on the limited geographical distribution of some of the organisms encoding these proteins and the intracellular localization of most of these proteins, it is unlikely that this would be the case, indicating that the immune responses should have been induced by infection with or exposure to JCPyV. The fact that there appears to be no correlation with JCPyV VP1 antibody levels, nor urinary viral load, indicates that these specific antibodies might be considered as a novel marker for infection with JCPyV.

## Material and methods

### Ethics statement

The Ethics Committee [“Commissie voor Medische Ethiek - ZiekenhuisNetwerk Antwerpen (ZNA) and the Ethics committee University Hospital Antwerp] approved the Protocols, and Informed consents, which were signed by all subjects.

### Healthy subject samples

For the screening study a total of 50 healthy subjects were recruited in Belgium, for the evaluation study a total of 204 healthy subjects (HS) were recruited in Belgium [14, 29, 45]. The demographic description of the HS populations is presented in Table 1. Plasma samples and urine samples were collected from all these HS and stored at −80°C until further processing.

**Table 1:** **Overview of subjects investigated**

Variable		Screening study (n = 50)	Evaluation study (n = 204)
Gender, n (%)			
	Male	22 (44%)	97 (48%)
	Female	28 (56%)	107 (52%)
	Age, median (Min-Max)	40.5 (23–59)	42 (19–66)
Race, ethnicity, n (%)			
	White	38 (76%)	201 (98.5%)
	Black	3 (6%)	0 (0%)
	Asian	8 (16%)	2 (1%)
	Other/unknown	1 (2%)	1 (0.5%)
JCPyV VP1 serology, n (%)			
	Positive	36 (72%)	156 (57%)
	Negative	14 (28%)	88 (43%)
JCPyV Viruria, n (%)			
	Positive*	13 (26%)	50 (25%)
	Negative	37 (74%)	154 (75%)
BKPyV VP1 serology, n (%)			
	Positive	n.d.	178 (88%)
	Negative	n.d.	24 (12%)
JCPyV_VP2_167-15mer serology, n (%)			
	Positive	32 (64%)	165 (81%)
	Negative	18 (36%)	39 (19%)

*All JCPyV viruric subjects were part of the JCPyV VP1 seropositive subgroup.

### JC polyomavirus viral load assay

Analysis of the urinary viral load was performed as described previously [14].

### JC polyomavirus VP1 serology assay

The anti-JCPyV antibody assay was performed as described earlier [45]. Samples were considered positive if OD values were higher than 2-fold the OD value of the blank sample (i.e. log_2_ test/ctrl > 1).

### BK polyomavirus VP1 serology assay

The anti-JCPyV antibody assay was performed identical to the method described for JCPyV VP1 with the exception that microtiter plates were coated with baculovirus expressed BKPyV VP1 capsid protein (Eurogentec, Belgium). Samples were considered positive if OD values were higher than 2-fold the OD value of the blank sample (i.e. log_2_ test/ctrl > 1).

### JC polyomavirus VP2 peptide microarrays

Peptide microarrays were performed as part of the work that was described earlier [29]. For this study only the peptides from JCPyV VP2 were included for further detailed analysis.

### Molecular modeling of JCPyV_VP2_154_36-mer

Molecular modeling was carried out using the program PEP-FOLD (web server http://mobyle.rpbs.univ-paris-diderot.fr/cgi-bin/portal.py#forms::PEP-FOLD, [46, 47]). The peptide sequence of JCPyV_VP2_154_36-mer (GPSLFSTISQAFWNLVRDDLPALTSQEIQRRTQK) was submitted to the server for peptide folding and resulting structure was visualized using Jmol.

### Synthetic peptides

Biotinylated synthetic peptides were synthesized by standard procedures and purchased from JPT Innovative Peptide Solutions (Berlin, Germany Peptide serology assay). A list of all peptide sequences used in this study is provided in Additional file 2: Table S2.

### Peptide serology assays

For determination of peptide specific plasma antibody levels peptide ELISA was developed and set up as follows. Ninety-six well flat bottom plates (Nunc C96 Maxisorp, VWR) were coated with 100 μL of 1 μg/mL Streptavidin from *Streptomyces avidinii* (Sigma) in Dulbecco’s PBS without calcium and magnesium (Gibco). The plates were incubated at 4°C for 16 hours. The plates were rinsed once with 200 μL PBS + 0.05% Tween-20 (washing buffer) and blocked for 1 hour at 37°C with 200 μL of 10-fold diluted Blocker Casein (Pierce) in PBS (blocking solution). Upon removal of the blocking solution, plates were incubated with continuous shaking for 2 hours at room temperature with 100 μL of the selected biotinylated peptide, which were diluted at 1 μg/mL in blocking solution. The plates were rinsed 3 times with washing buffer, thereby eliminating unbound peptide. Then, the different wells were covered with 100 μL of human plasma samples, diluted 1:200 in blocking solution. Each sample was analyzed in duplicate. In control wells, blocking solution was added instead. The plate was incubated at 37°C for 1 hour. After incubation, a new triple rinsing cycle was repeated as described above. Then, the secondary antibody solution was added to each well. The solution contained an affinity purified Donkey anti-human IgG (H + L) peroxidase conjugate (Jackson ImmunoResearch) diluted 1:10,000 in blocking solution. The reaction mixture was incubated at 37°C for 1 hour. At the end of the incubation period, the plates were rinsed 3 times with washing buffer and treated with 100 μL SureBlue™ TMB Microwell Peroxidase Substrate (KPL). After 10 minutes of incubation the colorimetric reaction was stopped with 100 μL 1 N HCl. The plate was then read by the spectrophotometer (SpectraMax, Molecular Devices) at a wavelength (λ) of 450 nm. Samples were considered positive if OD values were higher than 2-fold the OD value of the blank sample (i.e. log_2_ test/ctrl > 1).

### Statistical analysis

Differences in antibody levels between groups were assessed using a Mann-Whitney test. Differences between groups were considered statistically significant at *P* < 0.05. Correlation between different parameters was analyzed using linear regression. *P*-value was calculated to determine whether slope was significantly non-zero and strength of correlation was determined using r-value. All statistical analyses were performed using GraphPad Prism v 5.04.

## Electronic supplementary material

Additional file 1: Table S1: Proteins identified upon BLAST analysis of JCV_VP2_171-11mer. (DOCX 19 KB)

Additional file 2: Table S2: Sequences of peptides used in this study. (DOCX 21 KB)

## Abbreviations

(PML): Progressive multifocal leukoencephalopathy
(JCPyV): JC polyomavirus
(BKPyV): BK polyomavirus
(MS): Multiple sclerosis
(ELISA): Enzyme-linked immunosorbent assay
(OD): Optical density
(HSs): Healthy subjects.

## References

[1] Frisque RJ, Bream GL, Cannella MT (1984). Human polyomavirus JC virus genome. J Virol.

[2] Padgett BL, Rogers CM, Walker DL (1977). JC virus, a human polyomavirus associated with progressive multifocal leukoencephalopathy: additional biological characteristics and antigenic relationships. Infect Immun.

[3] Berger JR, Major EO (1999). Progressive multifocal leukoencephalopathy. Semin Neurol.

[4] Padgett BL, Walker DL, ZuRhein GM, Eckroade RJ, Dessel BH (1971). Cultivation of papova-like virus from human brain with progressive multifocal leucoencephalopathy. Lancet.

[5] Dubois V, Dutronc H, Lafon ME, Poinsot V, Pellegrin JL, Ragnaud JM, Ferrer AM, Fleury HJ (1997). Latency and reactivation of JC virus in peripheral blood of human immunodeficiency virus type 1-infected patients. J Clin Microbiol.

[6] Andreoletti L, Dubois V, Lescieux A, Dewilde A, Bocket L, Fleury HJ, Wattre P (1999). Human polyomavirus JC latency and reactivation status in blood of HIV-1-positive immunocompromised patients with and without progressive multifocal leukoencephalopathy. Aids.

[7] Weissert R (2011). Progressive multifocal leukoencephalopathy. J Neuroimmunol.

[8] Gasparovic ML, Gee GV, Atwood WJ (2006). JC virus minor capsid proteins Vp2 and Vp3 are essential for virus propagation. J Virol.

[9] Shishido-Hara Y, Ichinose S, Higuchi K, Hara Y, Yasui K (2004). Major and minor capsid proteins of human polyomavirus JC cooperatively accumulate to nuclear domain 10 for assembly into virions. J Virol.

[10] Giorda KM, Raghava S, Zhang MW, Hebert DN (2013). The viroporin activity of the minor structural proteins VP2 and VP3 is required for SV40 propagation. J Biol Chem.

[11] Knowles WA, Pipkin P, Andrews N, Vyse A, Minor P, Brown DW, Miller E (2003). Population-based study of antibody to the human polyomaviruses BKV and JCV and the simian polyomavirus SV40. J Med Virol.

[12] Lee P, Plavina T, Castro A, Berman M, Jaiswal D, Rivas S, Schlain B, Subramanyam M (2013). A second-generation ELISA (STRATIFY JCV DxSelect) for detection of JC virus antibodies in human serum and plasma to support progressive multifocal leukoencephalopathy risk stratification. J Clin Virol.

[13] Major EO (2010). Progressive multifocal leukoencephalopathy in patients on immunomodulatory therapies. Annu Rev Med.

[14] Van Loy T, Thys K, Tritsmans L, Stuyver LJ (2013). Quasispecies analysis of JC virus DNA present in urine of healthy subjects. PLoS One.

[15] Padgett BL, Walker DL (1973). Prevalence of antibodies in human sera against JC virus, an isolate from a case of progressive multifocal leukoencephalopathy. J Infect Dis.

[16] Taguchi F, Kajioka J, Miyamura T (1982). Prevalence rate and age of acquisition of antibodies against JC virus and BK virus in human sera. Microbiol Immunol.

[17] Walker DL, Padgett BL (1983). The epidemiology of human polyomaviruses. Prog Clin Biol Res.

[18] Randhawa P, Viscidi R, Carter JJ, Galloway DA, Culp TD, Huang C, Ramaswami B, Christensen ND (2009). Identification of species-specific and cross-reactive epitopes in human polyomavirus capsids using monoclonal antibodies. J Gen Virol.

[19] Viscidi RP, Clayman B (2006). Serological cross reactivity between polyomavirus capsids. Adv Exp Med Biol.

[20] Dominguez-Mozo MI, Garcia-Montojo M, De Las HV, Garcia-Martinez A, Arias-Leal AM, Casanova I, Arroyo R, Alvarez-Lafuente R (2013). Anti-JCV antibodies detection and JCV DNA levels in PBMC, serum and urine in a cohort of Spanish Multiple Sclerosis patients treated with natalizumab. J Neuroimmune Pharmacol.

[21] Plavina T, Berman M, Njenga M, Crossman M, Lerner M, Gorelik L, Simon K, Schlain B, Subramanyam M (2012). Multi-site analytical validation of an assay to detect anti-JCV antibodies in human serum and plasma. J Clin Virol.

[22] Warnke C, Pawlita M, Dehmel T, Posevitz-Fejfar A, Hartung HP, Wiendl H, Kieseier BC, Adams O (2013). An assay to quantify species-specific anti-JC virus antibody levels in MS patients. Mult Scler.

[23] Kean JM, Rao S, Wang M, Garcea RL (2009). Seroepidemiology of human polyomaviruses. PLoS Pathog.

[24] Moens U, Van Ghelue M, Song X, Ehlers B (2013). Serological cross-reactivity between human polyomaviruses. Rev Med Virol.

[25] Food-and-Drug-Administration (2012). Anti- John Cunningham Virus (JCV) antibodies measured by Enzyme Linked Immunosorbent Assay (ELISA); 510(k) Number: K112394.

[26] Zhang NN, Zhao LQ, Qian Y, Zhu RN, Deng J, Wang F, Sun Y, Liu LY (2013). Common WU polyomavirus infection in a Beijing population indicated by surveillance for serum IgG antibody against capsid protein VP2. World J Pediatr.

[27] Corallini A, Mazzoni E, Taronna A, Manfrini M, Carandina G, Guerra G, Guaschino R, Vaniglia F, Magnani C, Casali F, Dolcetti R, Palmonari C, Rezza G, Martini F, Barbanti-Brodano G, Tognon MG (2012). Specific antibodies reacting with simian virus 40 capsid protein mimotopes in serum samples from healthy blood donors. Hum Immunol.

[28] Mueller K, Schachtner T, Sattler A, Meier S, Friedrich P, Trydzenskaya H, Hinrichs C, Trappe R, Thiel A, Reinke P, Babel N (2011). BK-VP3 as a new target of cellular immunity in BK virus infection. Transplantation.

[29] Stuyver LJ, Verbeke T, Van Loy T, Van Gulck E, Tritsmans L (2013). An antibody response to human polyomavirus 15-mer peptides is highly abundant in healthy human subjects. Virol J.

[30] Cunningham BC, Wells JA (1993). Comparison of a structural and a functional epitope. J Mol Biol.

[31] Cunningham BC, Wells JA (1989). High-resolution epitope mapping of hGH-receptor interactions by alanine-scanning mutagenesis. Science.

[32] Zheng HY, Sugimoto C, Hasegawa M, Kobayashi N, Kanayama A, Rodas A, Mejia M, Nakamichi J, Guo J, Kitamura T, Yogo Y (2003). Phylogenetic relationships among JC virus strains in Japanese/Koreans and Native Americans speaking Amerind or Na-Dene. J Mol Evol.

[33] Altschul SF, Gish W, Miller W, Myers EW, Lipman DJ (1990). Basic local alignment search tool. J Mol Biol.

[34] Egli A, Infanti L, Dumoulin A, Buser A, Samaridis J, Stebler C, Gosert R, Hirsch HH (2009). Prevalence of polyomavirus BK and JC infection and replication in 400 healthy blood donors. J Infect Dis.

[35] Antonsson A, Green AC, Mallitt KA, O’Rourke PK, Pawlita M, Waterboer T, Neale RE (2010). Prevalence and stability of antibodies to the BK and JC polyomaviruses: a long-term longitudinal study of Australians. J Gen Virol.

[36] Stolt A, Sasnauskas K, Koskela P, Lehtinen M, Dillner J (2003). Seroepidemiology of the human polyomaviruses. J Gen Virol.

[37] Frohman EM, Monaco MC, Remington G, Ryschkewitsch C, Jensen PN, Johnson K, Perkins M, Liebner J, Greenberg B, Monson N, Frohman TC, Douek D, Major EO (2014). JC virus in CD34+ and CD19+ cells in patients with multiple sclerosis treated with natalizumab. JAMA Neurol.

[38] Major EO, Frohman E, Douek D (2013). JC viremia in natalizumab-treated patients with multiple sclerosis. N Engl J Med.

[39] Major EO, Frohman E, Douek D (2013). More on JC viremia in natalizumab-treated patients with multiple sclerosis. N Engl J Med.

[40] Berger JR, Houff SA, Gurwell J, Vega N, Miller CS, Danaher RJ (2013). JC virus antibody status underestimates infection rates. Ann Neurol.

[41] Diotti RA, Mancini N, Clementi N, Sautto G, Moreno GJ, Criscuolo E, Cappelletti F, Man P, Forest E, Remy L, Giannecchini S, Clementi M, Burioni R (2014). Cloning of the first human anti-JCPyV/VP1 neutralizing monoclonal antibody: epitope definition and implications in risk stratification of patients under natalizumab therapy. Antivir Res.

[42] Streckert HJ, Sommerfeld HJ, Morgenroth K, Werchau H (1992). Recognition of SV40-VP2 in the infected cell by antipeptide antibodies. Arch Virol.

[43] Chen XS, Stehle T, Harrison SC (1998). Interaction of polyomavirus internal protein VP2 with the major capsid protein VP1 and implications for participation of VP2 in viral entry. EMBO J.

[44] Burkert O, Kressner S, Sinn L, Giese S, Simon C, Lilie H (2014). Biophysical characterization of polyomavirus minor capsid proteins. Biol Chem.

[45] Lagatie O, Van Loy T, Tritsmans L, Stuyver LJ (2014). Circulating human microRNAs are not linked to JC polyomavirus serology or urinary viral load in healthy subjects. Virol J.

[46] Alland C, Moreews F, Boens D, Carpentier M, Chiusa S, Lonquety M, Renault N, Wong Y, Cantalloube H, Chomilier J, Hochez J, Pothier J, Villoutreix BO, Zagury JF, Tuffery P (2005). RPBS: a web resource for structural bioinformatics. Nucleic Acids Res.

[47] Neron B, Menager H, Maufrais C, Joly N, Maupetit J, Letort S, Carrere S, Tuffery P, Letondal C (2009). Mobyle: a new full web bioinformatics framework. Bioinformatics.

