# Supplementary material for: Antibodies reacting with JCPyV_VP2 _167-15mer as a novel serological marker for JC polyomavirus infection
Source: Virol J. 2014 Oct 1;11:174. doi: 10.1186/1743-422X-11-174 (PMC4194363; doi:10.1186/1743-422X-11-174)
Supplement: Supplementary file 1 — Additional file 1: Table S1: Proteins identified upon BLAST analysis of JCV_VP2_171-11mer. (DOCX 19 KB) [file 12985_2014_2502_MOESM1_ESM.docx]

**Additional file 1: Table S1. Proteins identified upon BLAST analysis of JCV_VP2_171-11mer.** Mismatches are underlined in the respective sequences. Synthetic peptides were prepared including the 4 upstream amino acids (indicated in grey) and used for determination of **plasma antibody reactivity of plasma samples from 3 HSs (diluted 1:200) with high immunoreactivity against the JCPyV_VP2_167-15mer.**

| **Organism** | **Protein name** | **Sequence** | **#mm*** |
| --- | --- | --- | --- |
| ***Viridae*** |  |  |  |
| JCV | VP2/VP3 | NLVRDDLPALTSQEI | 0/0 |
| SA12 | VP2/VP3 | HLVRDDLP**R**LTSQEI | 1/2 |
| BKV | VP2/VP3 | HVIRDD**I**P**SI**TSQE**L** | 4/7 |
| ***Bacteria*** |  |  |  |
| Psychrobacter sp. G | muramoyltetrapeptide carboxypeptidase | QLTKDDLP**T**ALTSQE**A** | 2/5 |
| Ruminococcus lactaris | hypothetical protein | EKQTDD**S**LP**S**LTSQ**D**I | 3/7 |
| Lachnospiraceae | hypothetical protein | EKQTDD**S**LP**S**LTSQ**D**I | 3/7 |
| Coprococcus comes | hypothetical protein | EKQTDD**S**LP**S**LTSQ**D**I | 3/7 |
| Clostridium termitidis | pyruvate kinase | PGAD**IR**LPALTSQ**D**I | 3/7 |
| Escherichia coli | phage tail protein E | VIPR**ISS**PALTSQEI | 3/6 |
| Vibrio proteolyticus | hypothetical protein | TAVR**A**DLPAL**SG**QEI | 3/5 |
| Rhodococcus sp. AW25M09 | putative cobalamin adenosyltransferase | DEFNDDLPALTS**FIL** | 3/7 |
| Achromobacter xylosoxidans | hypothetical protein | RVAPDDLPALTS**LAA** | 3/7 |
| Achromobacter piechaudii | hypothetical protein | RRDPDDLPALTS**LAA** | 3/7 |
| Nocardiopsis baichengensis | galactose-1-phosphate uridylyltransferase | HRKV**P**DLPALT**P**QE**R** | 3/7 |
| Dyella japonica | acetylglutamate kinase | AVLRDDLPALTS**SLT** | 3/6 |
| Frateuria aurantia DSM 6220 | acetylglutamate kinase | AVLRDDLPALTS**SLS** | 3/6 |
| Micrococcus luteus | isopropylmalate isomerase | LASP**S**DLPALT**P**QE**A** | 3/7 |
| Pseudomonas psychrophila | hypothetical protein | KRISDDLPALTS**IYV** | 3/7 |
| Pseudomonas chloritidismutans | glycosyl transferase family 51 | LCRPDDLPALTS**GML** | 3/7 |
| Pseudomonas stutzeri CCUG 29243 | membrane carboxypeptidase | LCRPDDLPALTS**GML** | 3/7 |
| Sphingobacterium sp. 21 | hypothetical protein | RANA**VN**LPA**F**TSQEI | 3/7 |
| Acidocella sp. MX-AZ02 | RNA polymerase sigma70 | EPVPDDLPAL**-**SQE**T** | 2/5 |
| Paenibacillus sp. JDR-2 | oligoendopeptidase | QYVMDDLPAL**-**SQE**Y** | 2/5 |
| Spirosoma linguale DSM 74 | anti-FecI sigma factor FecR | TARH**S**DLPAL**S**S**D**EI | 3/7 |
| Beggiatoa alba | hypothetical protein | NLQE**MT**LPALT**R**QEI | 3/5 |
| Leptospira kirschneri | leucine rich repeat protein | TLNLDD**I**PAL**K**SQE**K** | 3/6 |
| Pasteurella multocida | hypothetical protein | VELR**YM**LPALT**E**QEI | 3/6 |
| ***Animalia*** |  |  |  |
| Ixodes scapularis | perixosomal biogenesis factor, putative | QTSG**GV**LPALTSQEI | 2/6 |
| Caenorhabditis briggsae | CBR-SET-11 protein | VREQD**E**LPAL**-**SQEI | 2/6 |
| Drosophila pseudoobscura pseudoobscura | GA14246 | LTPED**E**LPAL**N**SQE**E** | 3/7 |
| Drosophila persimilis | GL26700 | LTPED**E**LPAL**N**SQE**E** | 3/7 |
| Tupaia chinensis | tubby-related protein 4 | SEDEDDLPALTS**SNQ** | 3/7 |
| ***Human*** |  |  |  |
| Homo sapiens | coiled-coil domain-containing protein 160 | TCSTD**N**LPAL**LR**Q**D**I | 4/8 |
| ***Other*** |  |  |  |
| Naegleria gruberi | WD40 repeat domain-containing protein | MTTNDDL**A**ALT**T**QEI | 2/6 |
| Brachypodium distachyon | probable E3 ubiquitin-protein ligase ARI1-like | KVLT**R**D**V**PALT**N**QEI | 3/7 |

* number of mismatches in 11mer / 15mer
