# Supplementary material for: Antibodies reacting with JCPyV_VP2 _167-15mer as a novel serological marker for JC polyomavirus infection
Source: Virol J. 2014 Oct 1;11:174. doi: 10.1186/1743-422X-11-174 (PMC4194363; doi:10.1186/1743-422X-11-174)
Supplement: Supplementary file 2 — Additional file 2: Table S2: Sequences of peptides used in this study. (DOCX 21 KB) [file 12985_2014_2502_MOESM2_ESM.docx]

**Additional file 1 Sequences of peptides used in this study**

| **Figure 1A** | |
| --- | --- |
| 1 | MGAALALLGDLVATV |
| 1 | MGAAVALLGDLVATV |
| 2 | GAALALLGDLVATVS |
| 2 | GAAVALLGDLVATVS |
| 6 | ALLGDLVATVSEAAA |
| 10 | DLVATVSEAAAATGF |
| 23 | GFSVAEIAAGEAAAT |
| 31 | AGEAAATIEVEIASL |
| 35 | AATIEVEIASLATVE |
| 39 | EVEIASLATVEGITT |
| 43 | ASLATVEGITTTSEA |
| 47 | TVEGITTTSEAIAAI |
| 54 | TSEAIAAIGLTPETY |
| 58 | IAAIGLTPETYAVIT |
| 62 | GLTPETYAVITGAPG |
| 66 | ETYAVITGAPGAVAG |
| 70 | VITGAPGAVAGFAAL |
| 74 | APGAVAGFAALVQTV |
| 78 | VAGFAALVQTVTGGS |
| 82 | AALVQTVTGGSAIAQ |
| 86 | QTVTGGSAIAQLGYR |
| 86 | QTVTGGSAIAQVGYR |
| 90 | GGSAIAQLGYRFFAD |
| 90 | GGSAIAQVGYRFFAD |
| 94 | IAQLGYRFFADWDHK |
| 94 | IAQVGYRFFADWDHK |
| 98 | GYRFFADWDHKVSTV |
| 104 | DWDHKVSTVGLFQQP |
| 108 | KVSTVGLFQQPAMAL |
| 108 | KVSTVGLFQQPVMAL |
| 112 | VGLFQQPAMALQLFN |
| 112 | VGLFQQPVMALQLFN |
| 116 | QQPAMALQLFNPEDY |
| 116 | QQPVMALQLFNPEDY |
| 120 | MALQLFNPEDYYDIL |
| 124 | LFNPEDYYDILFPGV |
| 128 | EDYYDILFPGVNAFV |
| 132 | DILFPGVNAFVNNIH |
| 136 | PGVNAFVNNIHYLDP |
| 140 | AFVNNIHYLDPRHWG |
| 147 | YLDPRHWGPSLFSTI |
| 151 | RHWGPSLFSTISQAF |
| 155 | PSLFSTISQAFWNLV |
| 159 | STISQAFWNLVRDDL |
| 163 | QAFWNLVRDDLPSLT |
| 167 | NLVRDDLPSLTSQEI |
| 167 | NLVRDDLPALTSQEI |
| 171 | DDLPSLTSQEIQRRT |
| 176 | LTSQEIQRRTQKLFV |
| 180 | EIQRRTQKLFVESLA |
| 184 | RTQKLFVESLARFLE |
| 188 | LFVESLARFLEETTW |
| 193 | LARFLEETTWAIVNS |
| 197 | LEETTWAIVNSPVNL |
| 201 | TWAIVNSPVNLYNYI |
| 205 | VNSPVNLYNYISDYY |
| 210 | NLYNYISDYYSRLSP |
| 214 | YISDYYSRLSPVRPS |
| 218 | YYSRLSPVRPSMVRQ |
| 222 | LSPVRPSMVRQVAQR |
| 226 | RPSMVRQVAQREGTY |
| 230 | VRQVAQREGTYISFG |
| 234 | AQREGTYISFGHSYT |
| 238 | GTYISFGHSYTQSID |
| 242 | SFGHSYTQSIDDADS |
| 246 | SYTQSIDDADSIQEV |
| 251 | IDDADSIQEVTQRLD |
| 255 | DSIQEVTQRLDLKTP |
| 259 | EVTQRLDLKTPNVQS |
| 263 | RLDLKTPNVQSGEFI |
| 269 | PNVQSGEFIEKSIAP |
| 273 | SGEFIEKSIAPGGAN |
| 277 | IEKSIAPGGANQRSA |
| 282 | APGGANQRSAPQWML |
| 286 | ANQRSAPQWMLPLLL |
| 307 | TPALEAYEDGPNKKK |
| 311 | EAYEDGPNKKKRRKE |
| 315 | DGPNKKKRRKEGPRA |
| 319 | KKKRRKEGPRASSKT |
| 323 | RKEGPRASSKTSYKR |
| 327 | PRASSKTSYKRRSRS |
| 330 | SSKTSYKRRSRSSRS |

| **Figure 1D** | |
| --- | --- |
| JCV_VP2_167-15mer | NLVRDDLPALTSQEI |
| BKV_VP2_167-15mer | HVIRDDIPSITSQEL |

| **Figure 3A** | |
| --- | --- |
| JCV_VP2_163-7mer | QAFWNLV |
| JCV_VP2_164-7mer | AFWNLVR |
| JCV_VP2_165-7mer | FWNLVRD |
| JCV_VP2_166-7mer | WNLVRDD |
| JCV_VP2_167-7mer | NLVRDDL |
| JCV_VP2_168-7mer | LVRDDLP |
| JCV_VP2_169-7mer | VRDDLPA |
| JCV_VP2_170-7mer | RDDLPAL |
| JCV_VP2_171-7mer | DDLPALT |
| JCV_VP2_172-7mer | DLPALTS |
| JCV_VP2_173-7mer | LPALTSQ |
| JCV_VP2_174-7mer | PALTSQE |
| JCV_VP2_175-7mer | ALTSQEI |
|  |  |
| JCV_VP2_163-9mer | QAFWNLVRD |
| JCV_VP2_164-9mer | AFWNLVRDD |
| JCV_VP2_165-9mer | FWNLVRDDL |
| JCV_VP2_166-9mer | WNLVRDDLP |
| JCV_VP2_167-9mer | NLVRDDLPA |
| JCV_VP2_168-9mer | LVRDDLPAL |
| JCV_VP2_169-9mer | VRDDLPALT |
| JCV_VP2_170-9mer | RDDLPALTS |
| JCV_VP2_171-9mer | DDLPALTSQ |
| JCV_VP2_172-9mer | DLPALTSQE |
| JCV_VP2_173-9mer | LPALTSQEI |
|  |  |
| JCV_VP2_163-11mer | QAFWNLVRDDL |
| JCV_VP2_164-11mer | AFWNLVRDDLP |
| JCV_VP2_165-11mer | FWNLVRDDLPA |
| JCV_VP2_166-11mer | WNLVRDDLPAL |
| JCV_VP2_167-11mer | NLVRDDLPALT |
| JCV_VP2_168-11mer | LVRDDLPALTS |
| JCV_VP2_169-11mer | VRDDLPALTSQ |
| JCV_VP2_170-11mer | RDDLPALTSQE |
| JCV_VP2_171-11mer | DDLPALTSQEI |
|  |  |
| JCV_VP2_163-13mer | QAFWNLVRDDLPA |
| JCV_VP2_164-13mer | AFWNLVRDDLPAL |
| JCV_VP2_165-13mer | FWNLVRDDLPALT |
| JCV_VP2_166-13mer | WNLVRDDLPALTS |
| JCV_VP2_167-13mer | NLVRDDLPALTSQ |
| JCV_VP2_168-13mer | LVRDDLPALTSQE |
| JCV_VP2_169-13mer | VRDDLPALTSQEI |
|  |  |
| JCV_VP2_167-15mer | NLVRDDLPALTSQEI |

| **Figure 3C** | |
| --- | --- |
| Wild type | NLVRDDLPSLTSQEI |
| N167A | ALVRDDLPSLTSQEI |
| L168 | NAVRDDLPSLTSQEI |
| V169 | NLARDDLPSLTSQEI |
| R170 | NLVADDLPSLTSQEI |
| D171 | NLVRADLPSLTSQEI |
| D172 | NLVRDALPSLTSQEI |
| L173 | NLVRDDAPSLTSQEI |
| P174 | NLVRDDLASLTSQEI |
| S175 | NLVRDDLPALTSQEI |
| L176 | NLVRDDLPSATSQEI |
| T177 | NLVRDDLPSLASQEI |
| S178 | NLVRDDLPSLTAQEI |
| Q179 | NLVRDDLPSLTSAEI |
| E180 | NLVRDDLPSLTSQAI |
| I181 | NLVRDDLPSLTSQEA |

| **Figure 4** | |
| --- | --- |
| NLVRDDLPALTSQEI |  |
| HLVRDDLPRLTSQEI |  |
| HVIRDDIPSITSQEL |  |
| QLTKDDLPTALTSQEA |  |
| EKQTDDSLPSLTSQDI |  |
| PGADIRLPALTSQDI |  |
| VIPRISSPALTSQEI |  |
| TAVRADLPALSGQEI |  |
| DEFNDDLPALTSFIL |  |
| RVAPDDLPALTSLAA |  |
| RRDPDDLPALTSLAA |  |
| HRKVPDLPALTPQER |  |
| AVLRDDLPALTSSLT |  |
| AVLRDDLPALTSSLS |  |
| LASPSDLPALTPQEA |  |
| KRISDDLPALTSIYV |  |
| LCRPDDLPALTSGML |  |
| RANAVNLPAFTSQEI |  |
| EPVPDDLPALSQET |  |
| QYVMDDLPALSQEY |  |
| TARHSDLPALSSDEI |  |
| NLQEMTLPALTRQEI |  |
| TLNLDDIPALKSQEK |  |
| VELRYMLPALTEQEI |  |
| QTSGGVLPALTSQEI |  |
| VREQDELPALSQEI |  |
| LTPEDELPALNSQEE |  |
| SEDEDDLPALTSSNQ |  |
| TCSTDNLPALLRQDI |  |
| MTTNDDLAALTTQEI |  |
| KVLTRDVPALTNQEI |  |
